# Meta-analysis of high-flow nasal cannula oxygen therapy versus non-invasive ventilation after invasive mechanical ventilation

**DOI:** 10.3389/fmed.2025.1594145

**Published:** 2025-09-25

**Authors:** Mailidan Maimaitiniyazi, Tuersunayi Yisimitila, Muyesaier Maimaitiniyazi, Meiheliya Maisuti, Ailifeire Aihaiti, Zhang Chenfei, Yilizhati Nijiati, Muyesai Nijiati

**Affiliations:** ^1^Emergency Center, People’s Hospital of Xinjiang Uygur Autonomous Region, Ürümqi, China; ^2^Xinjiang Medical University, Ürümqi, China

**Keywords:** HFNC, NIV, invasive mechanical ventilation, meta-analysis, respiratory failure

## Abstract

**Objective:**

To compare high-flow nasal cannula (HFNC) oxygen therapy and non-invasive ventilation (NIV) for patients after liberation from invasive mechanical ventilation, to assess whether HFNC is better than NIV at improving blood gases [PaO_2_, PaCO_2_, and oxygenation index (OI)], reducing re-intubation rates, pulmonary infections, mortality, and shortening the length of stay in intensive care unit (ICU), and to evaluate if HFNC is a feasible alternative to NIV for respiratory support.

**Methods:**

This meta-analysis included randomized controlled trials (RCTs) and non-RCTs (NRCTs) from PubMed, Web of Science, CNKI, and Wanfang for further assessment. Evaluation indexes included PaO_2_, PaCO_2_, OI, re-intubation rate, pulmonary infection rate, length of stay in ICU, and mortality rate.

**Results:**

HFNC showed higher PaO_2_ [MD = 2.95, 95%CI (2.23, 3.67), *p* < 0.00001], lower PaCO_2_ [MD = −3.04, 95%CI (−3.56, −2.52), *p* < 0.00001], higher OI [MD = 10.98, 95%CI (6.52, 15.45), *p* < 0.00001], lower re-intubation rate [OR = 0.45, 95%CI (0.33, 0.63), *p* < 0.00001], and shorter length of stay in ICU [MD = −6.15, 95%CI (−6.86, −5.44), *p* < 0.00001] compared to NIV. Additionally, no significant differences in pulmonary infection rate [OR = 0.57, 95%CI (0.29, 1.11), *p* = 0.10] or mortality [OR = 1.29, 95%CI (0.96, 1.72), *p* = 0.09] were observed between HFNC and NIV.

**Conclusion:**

HFNC can improve PaO_2_ and OI, reduce PaCO_2_, re-intubation rate, and length of stay in ICU, with no difference in pulmonary infection or mortality compared to NIV, supporting it as a viable clinical alternative for post-extubation respiratory support.

## 1 Introduction

Invasive mechanical ventilation has been recognized to be an important supportive measure for acute respiratory failure. However, complications such as asphyxia, dyspnea and hypoxemia during tracheal extubation after condition stabilization, when patients are ready for extubation, will increase the risk of pulmonary infection and prolong the length of stay in the hospital (1). It highlights the significance and requirement for one mode of non-invasive ventilation after invasive ventilation. As clinical practice advances, a series of research hotspots and controversial issues have emerged in the application of oxygen therapy. Currently, there are three non-invasive modalities to increase post-extubation oxygenation, including conventional oxygen therapy, high-flow nasal cannula (HFNC) oxygen therapy, and non-invasive ventilation (NIV) (2). Clinically, NIV is a relatively common therapeutic option for managing patients with respiratory failure, which can facilitate an effective relief of dyspnea, reduce arterial partial pressure of carbon dioxide (PaCO_2_) and increase arterial partial pressure of oxygen (PaO_2_) in patients. However, its therapeutic effect may be compromised by factors such as relatively high complication rate and poor tolerance in some patients (3).

It promotes the introduction of novel therapeutic approaches in the clinical setting. Among these, HFNC has been gradually concerned due to its significant effects (4). HFNC, with the functions of providing heating and humidification, was initially used for neonates or infants with respiratory distress (5). It can deliver oxygen at a gas flow rate of 0–60 L/min, an oxygen concentration of 21–100%, and a temperature of 37 °C. It can meet patients’ requirements for humidity and temperature simultaneously, thus alleviating the damage to the tracheal mucosa caused by humidity and temperature during conventional oxygen therapy (6).

HFNC offers better comfort than NIV, which can improve patients’ compliance to cooperate with treatment (7, 8), consequently holding significant potential for broader clinical application. However, it is still inconclusive regarding the clinical efficacy of HFNC revealed by current evidence. Therefore, based on the retrieval of literature comparing HFNC with NIV in post-extubation patients, this study employed a systematic review and meta-analysis. Existing meta-analyses, despite investigation on the efficacy differences between HFNC and NIV (9, 10), possess significant limitations, such as primarily focusing on adult COPD patients. In contrast, this study included populations with multiple conditions and of diverse age groups (premature infants, lung transplant recipients, and post-cardiac surgery patients), covering a wider range of underlying diseases and patients. Significantly, this design in our study may address clinical scenarios not covered by prior research, which can benefit a better interpretation of heterogeneity encountered in real-world clinical practice, and enhance the robustness of conclusions through sensitivity analysis and based on a larger sample size. This study intended to evaluate whether HFNC is an effective and safe therapeutic modality, whether it offers advantages over NIV, and whether it can serve as an alternative strategy to NIV, thereby providing evidence-based support for the clinical application of HFNC.

## 2 Data and methods

### 2.1 Study selection

This study focused on the inclusion of randomized controlled trials (RCTs) or non-RCTs (NRCTs) investigating sequential therapy with HFNC or NIV in patients following liberation from invasive mechanical ventilation. These trials enrolled patients with: chronic obstructive pulmonary disease (COPD) complicated by type II respiratory failure, acute exacerbation of COPD, post-lung transplantation, acute respiratory failure, postoperative hypoxemia, and post-extubation preterm infants. Eligible studies compared HFNC with NIV reporting at least one of the following outcomes were included: ① PaO_2_, ② PaCO_2_, ③ oxygenation index (OI), ④ re-intubation rate, ⑤ pulmonary infection rate, ⑥ length of stay in intensive care unit (ICU), and ⑦ mortality rate. In addition to the inclusion criteria, this study also collected patients’ characteristics, such as age, gender, sample size, and features of the intervention and control groups. We excluded studies involving: (1) patients without a history of intubation therapy, (2) literature with incomplete raw data, (3) duplicate publications, and (4) literature in languages other than Chinese or English. Ethical approval was not required for this systematic review as it did not utilize patient-level data.

### 2.2 Electronic search strategy

We searched for studies comparing the sequential efficacy of HFNC and NIV in patients following invasive mechanical ventilation from PubMed, Web of Science, CNKI, and Wanfang, from database inception to February 2025. Search terms included: (“Non-Positive Pressure Ventilation” OR “NPPV” OR “Non-Invasive Positive Pressure Ventilation” OR “NIPPV” OR “Non-invasive ventilation” OR “NIV” AND “High-flow oxygen therapy” OR “High-flow nasal cannula” OR “HFNC”).

### 2.3 Data collection and analysis

Through management using EndNote X9, the retrieved literature was independently screened by reviewing corresponding titles and abstracts by two authors. With the exclusion of literature that was obviously not relevant to our research, the remaining articles underwent full-text review based on the predefined inclusion and exclusion criteria. The methodological quality and extracted data were assessed by two researchers independently; and discrepancies were resolved through discussion or by a third reviewer. Corresponding authors were contacted for complete raw data if necessary.

### 2.4 Literature quality assessment

The quality of the included eligible studies was independently assessed by two researchers using the Cochrane Handbook for Systematic Reviews of Interventions and Review Manager (RevMan) software version 5.3. Evaluation criteria encompassed: random sequence generation, allocation concealment, blinding of participants and personnel, blinding of outcome assessment, incomplete outcome data, selective reporting, and other potential sources of bias. Based on the risk of bias scores, literature quality was categorized as low risk (≥5 points), moderate risk (3–4 points), or high risk (1–2 points) (11).

### 2.5 Statistical analysis

Meta-analysis was conducted using RevMan 5.3, with mean difference (MD) and odds ratio (OR) for continuous variables and dichotomous variables, respectively, both reported with 95% confidence intervals (CI). According to the assessment of heterogeneity using *I*^2^ and *p*-values, fixed-effects model and random-effects model were, respectively, adopted when there was low (*p* > 0.1 and *I*^2^ < 50%) and high heterogeneity (*p* ≤ 0.1 and *I*^2^ ≥ 50%) (12), with sensitivity analysis employed to identify heterogeneity sources. Sensitivity analysis was performed by removing individual studies to assess their impact on the overall results. Finally, forest and fennel plots were generated to indicate the meta-analysis results, and assess publication bias, respectively. Statistical significance was set at *p* < 0.05.

## 3 Results

### 3.1 Literature search results and flow chart of literature screening flow

**Figure 1:**
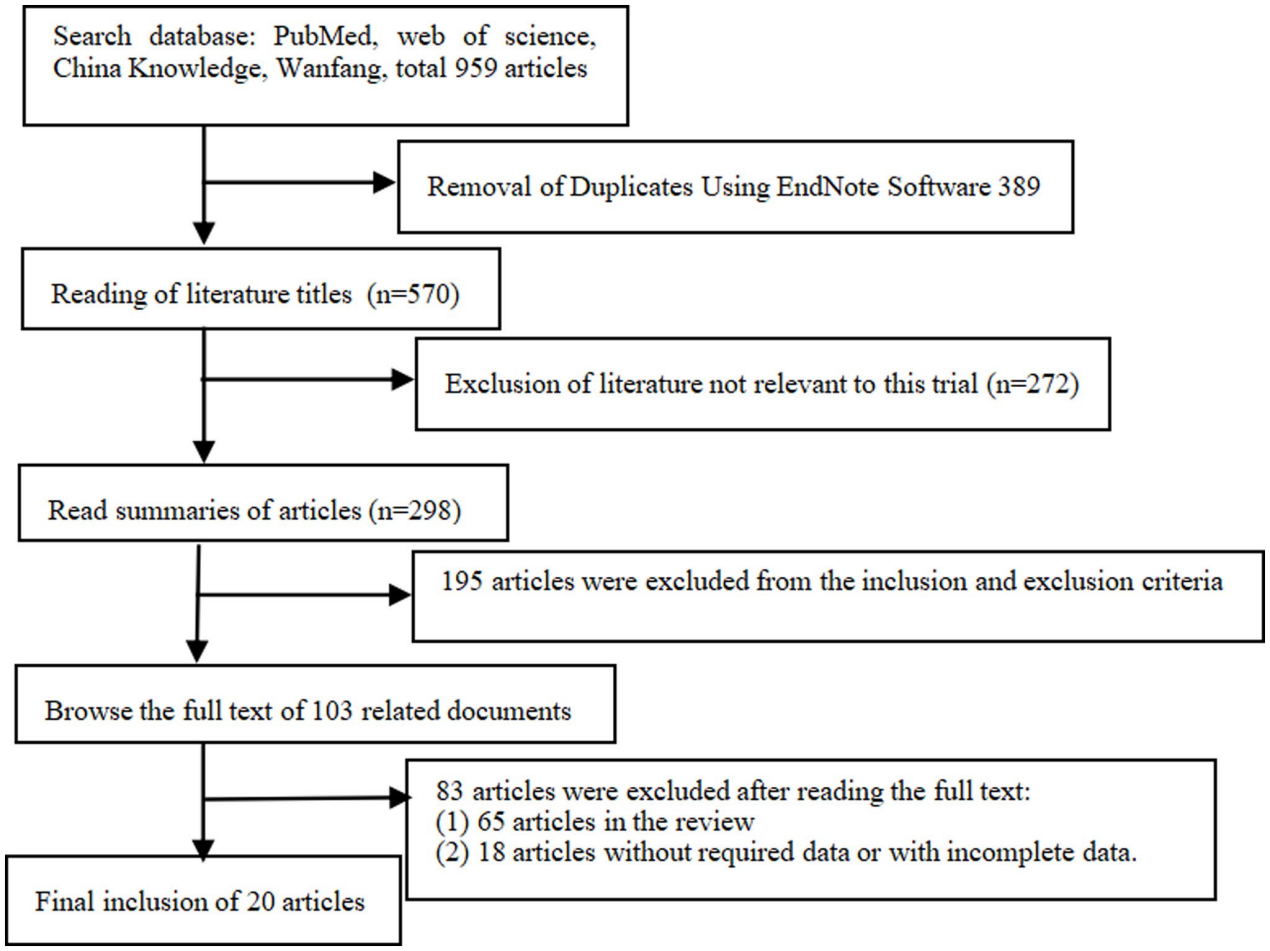
Literature screening flow chart.

Initially, 959 articles were retrieved from PubMed, Web of Science, CNKI, and Wanfang databases. Finally, 20 (13–32) studies [18 RCTs (13–30) and 2 NRCTs (31, 32)] were included for meta-analysis after removing 389 duplicates, 272 irrelevant articles, 195 non-compliant studies, 65 reviews, and 18 articles with incomplete data. These studies involved 3,159 patients, including 1,447 in the HFNC group and 1,712 in the NIV group. The screening process is detailed in Figure 1, and the baseline characteristics of the included studies are summarized in Table 1.

**Table 1:** Baseline characteristics of the included studies.

Author	Country of publication	Type of study	Sample size (cases)		Sex (M/F)		Age		Primary disease	Outcome indicator
			HNFC Group	NIV Group	HNFC group	NIV group	HNFC group	NIV group		
Li (13)	China	RCT	36	36	21/15	19/17	(65.32 ± 10.88)	(65.28 ± 11.91)	COPD combined with type II respiratory failure	①②③④⑥
Gao et al. (14)	China	RCT	206	276	37/69	188/88	(62.34 ± 5.54)	(62.57 ± 4.10)	Post-operative cardiac hypoxemia	①②④⑤⑥
Huang et al. (15)	China	RCT	28	32	22/6	23/9	(64.50 ± 3.21)	(65.28 ± 4.87)	Acute exacerbation of COPD	②③④⑥
Yuan (16)	China	RCT	56	44	31/25	25/19	(49.01 ± 9.00)	(52.00 ± 8.02)	Post Lung Transplantation	④⑥
Huang et al. (17)	China	RCT	150	151	97/53	91/60	(72.87 ± 6.97)	(72.92 ± 7.11)	Acute respiratory failure	①②⑥⑦
Yu et al. (18)	China	RCT	18	17	13/5	11/6	(75.00 ± 5.10)	(71.00 ± 6.50)	Acute exacerbation of COPD	①②④⑥
Han et al. (19)	China	RCT	55	55	36/19	35/20	(65.70 ± 7.30)	(66.20 ± 6.80)	Acute exacerbation of COPD	①②④⑥⑦
Xu et al. (20)	China	RCT	43	43	26/17	28/15	(65.80 ± 4.60)	(65.20 ± 5.30)	COPD	①②④⑥
Yue (21)	China	RCT	30	30	20/10	21/9	(78.34 ± 2.16)	(78.27 ± 2.33)	COPD	①②③④⑥
Zhang (22)	China	RCT	40	40	27/13	25/15	(67.41 ± 18.92)	(68.15 ± 16.22)	Acute exacerbation of COPD	①②③④⑥
Li et al. (23)	China	RCT	24	21	17/7	16/5	(52.30 ± 18.50)	(48.91 ± 22.40)	Post-operative hypoxemia	②③④⑥⑦
Wang et al. (24)	China	RCT	24	24	15/9	13/11	(51.30 ± 11.60)	(53.80 ± 9.30)	Post Lung Transplantation	③④⑤⑥⑦
Chen et al. (25)	China	RCT	48	46	30/18	29/17	(27.20 ± 2.80)	(27.50 ± 3.200)	Preterm infants after extubation	④
Uchiyama et al. (26)	Japan	RCT	176	196	91/85	97/99	(28.40 ± 3.00)	(28.20 ± 3.00)	Preterm infants after extubation	②④
Liu et al. (27)	USA	RCT	82	96	52/29	32/64	48~74	47~72	Sequential therapy after mechanical ventilation	④⑥⑦
Shi and Qiu (28)	China	RCT	60	60	32/28	36/24	64.64 ± 10.34	(64.64 ± 10.34)	AE-COPD combined with type II respiratory failure	①②③
Zheng and Wang (29)	China	RCT	47	47	27/20	25/22	60.57 ± 6.09	61.42 ± 6.17	Acute respiratory failure	②③④
Lu et al. (30)	China	RCT	49	48	28/21	30/18	67.88 ± 10.36	68.31 ± 9.62	AE-COPD combined with type II respiratory failure	①②⑦
Koga et al. (31)	Japan	NRCT	200	378	127/73	231/147	66–82	69–84	Acute respiratory failure	⑦
Chen et al. (32)	China	NRCT	75	72	43/32	38/34	70 ± 14	69 ± 16	Respiratory failure	①②④⑦

### 3.2 Results of literature quality evaluation

The quality of the 18 (13–30) RCTs included in our study was assessed using RevMan 5.3, showing high overall quality (Tables 2, 3). Meanwhile, the other 2 (31, 32) retrospective cohort studies were evaluated using the Newcastle-Ottawa Scale (NOS), both (13–32) of which revealed high quality and were suitable for Meta-analysis, as shown in Table 4.

**Table 2:** Evaluation on the methodological quality of the included studies.

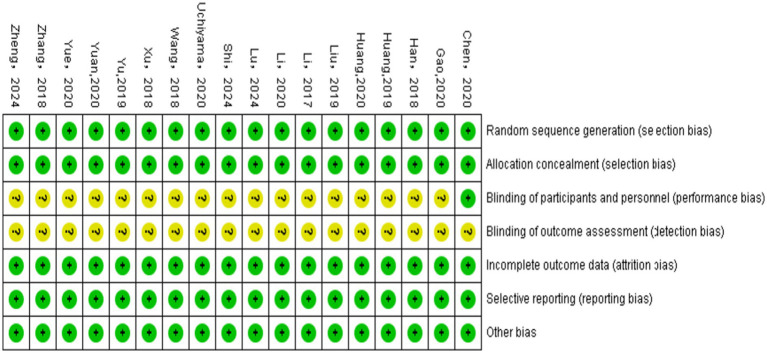

**Table 3:** Percentage of risk of bias.

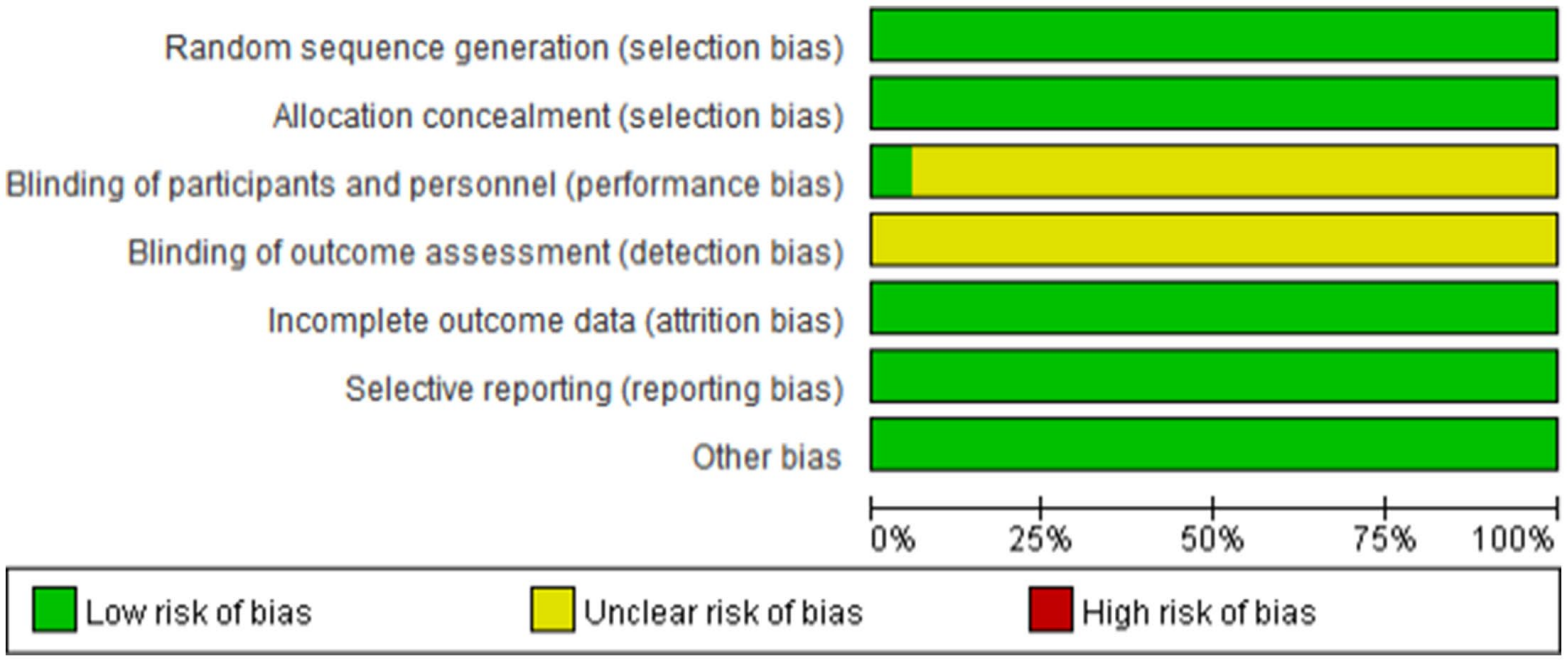

**Table 4:** Literature quality assessment.

First author/year of publication	Selection of study populations	Comparability of groups	Outcome measures	Total score	Quality ratings
Koga et al. (31), 2020	3	2	2	7	High quality
Chen et al. (32), 2023	3	2	2	7	High quality

The full score of the NOS is 9 points, with high-, moderate- and low-quality determined based on the score of ≥7, 5–6, and <5 points, respectively.

### 3.3 Outcomes of the meta-analysis

The comparative efficacy of HFNC versus NIV across the primary and secondary outcomes was rigorously evaluated. We now present the detailed meta-analytic results for each specific outcome measure.

#### 3.3.1 Differences in PaO_2_ levels between groups

**Figure 2:**
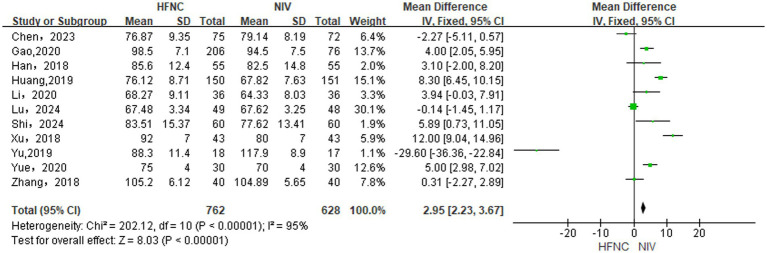
Forest plot of meta-analysis comparing PaO_2_ between HFNC group and NIV group.

Inter-group comparison of the difference in PaO_2_ levels incorporated 10 RCTs (13, 14, 17–22, 28, 30) and 1 NRCT (32). With the use of a random-effects model (*p* < 0.00001, *I*^2^ = 95%), HFNC significantly improved PaO_2_ over NIV [MD = 2.95, 95% CI (2.23, 3.67), *p* < 0.00001], as shown in Figure 2.

#### 3.3.2 Differences in PaCO_2_ levels between groups

**Figure 3:**
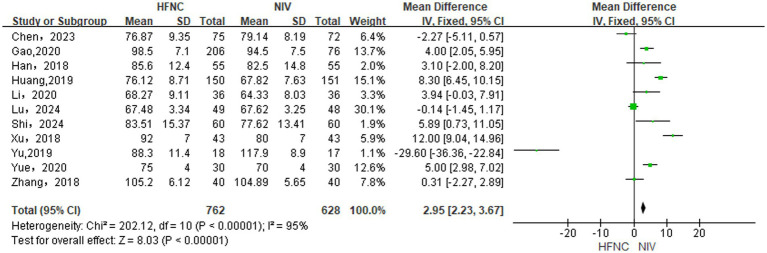
Forest plot of meta-analysis comparing PaCO_2_ between HFNC group and NIV group.

With the inclusion of 14 RCTs (13–15, 17–23, 26, 28–30) and 1 NRCT (32), a random-effects model (*p* < 0.00001, *I*^2^ = 95%) was used for analysis. Consequently, HFNC was superior to NIV in reducing PaCO_2_ [MD = −3.04, 95% CI (−3.56, −2.52), *p* < 0.00001], as shown in Figure 3.

#### 3.3.3 Differences in OI levels between groups

**Figure 4:**
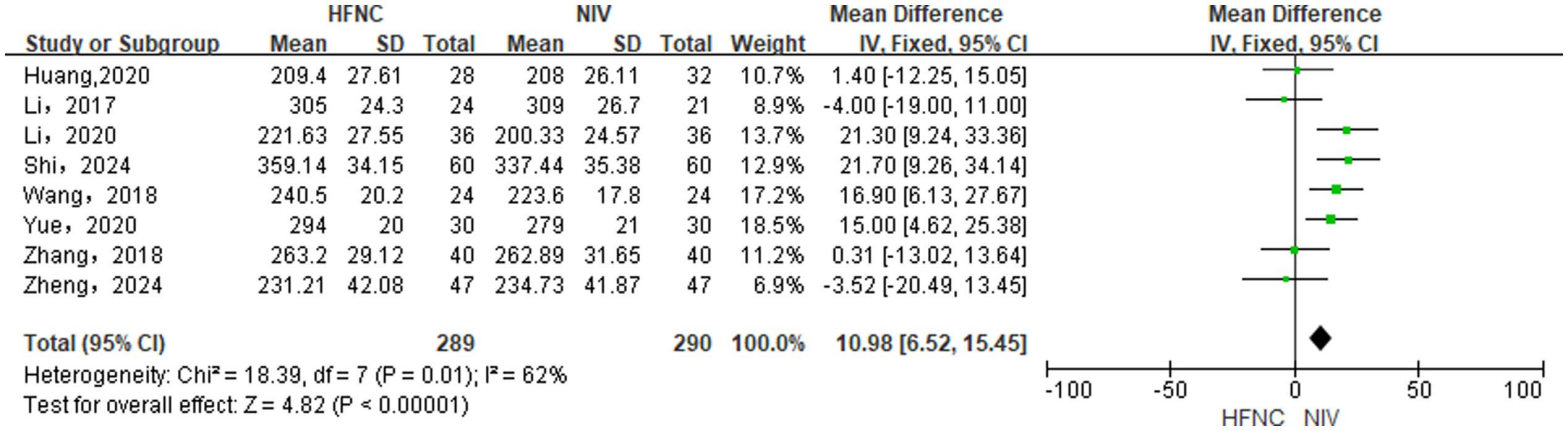
Forest plot of meta-analysis comparing OI between HFNC group and NIV group.

A random-effects model (*p* = 0.01, *I*^2^ = 62%) was used for comparing differences in OI levels between groups based on the inclusion of 8 RCTs (13, 15, 21–24, 28, 29). HFNC was superior to NIV in improving OI levels [MD = 10.98, 95%CI (6.52, 15.45), *p* < 0.00001], Figure 4.

#### 3.3.4 Difference in re-intubation rate between groups

**Figure 5:**
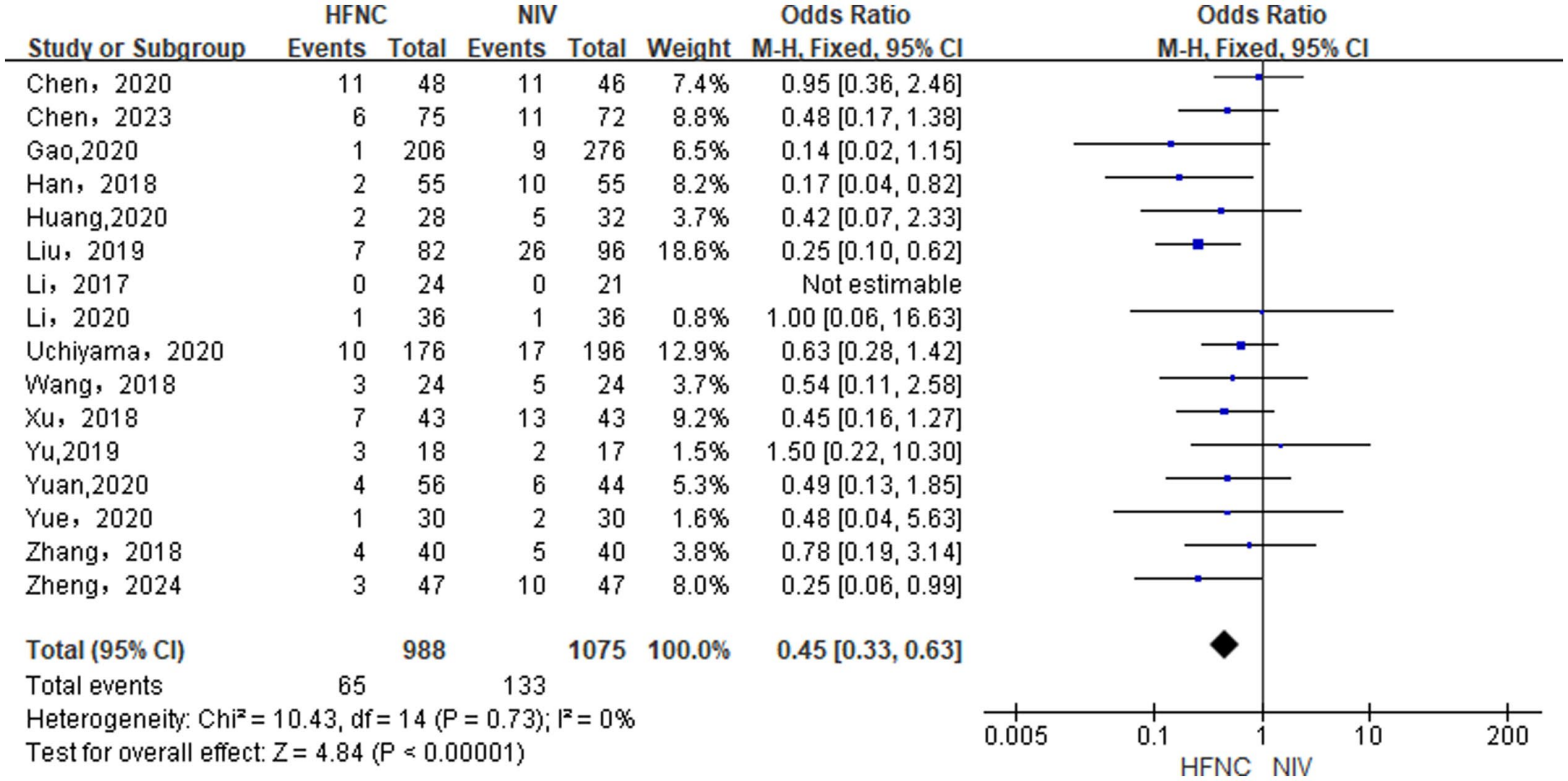
Forest plot of meta-analysis comparing re-intubation rate between HFNC group and NIV group.

Based on the inclusion of 15 RCTs (13–16, 18–27, 29) and 1 NRCT (32), analysis in a fixed-effects model (*p* = 0.73, *I*^2^ = 0%) showed that HFNC was superior to NIV in reducing re-intubation rate, [OR = 0.45, 95% CI (0.33, 0.63), *p* < 0.00001], as depicted in Figure 5.

#### 3.3.5 Differences in the pulmonary infection rate between groups

**Figure 6:**
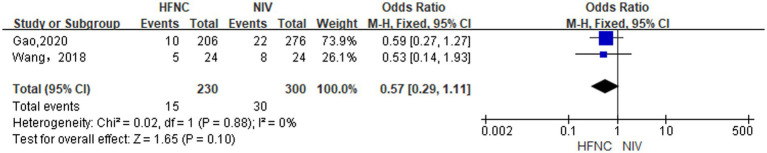
Forest plot of meta-analysis comparing the pulmonary infection rate between HFNC group and NIV group.

As revealed by the analysis in 2 RCTs (14, 24) using a fixed-effects model (*p* = 0.88, *I*^2^ = 0%), HFNC did not differ from NIV in reducing pulmonary infection rate [OR = 0.57, 95% CI (0.29, 1.11), *p* = 0.10], as shown in Figure 6.

#### 3.3.6 Differences in duration of stay in the ICU between groups

**Figure 7:**
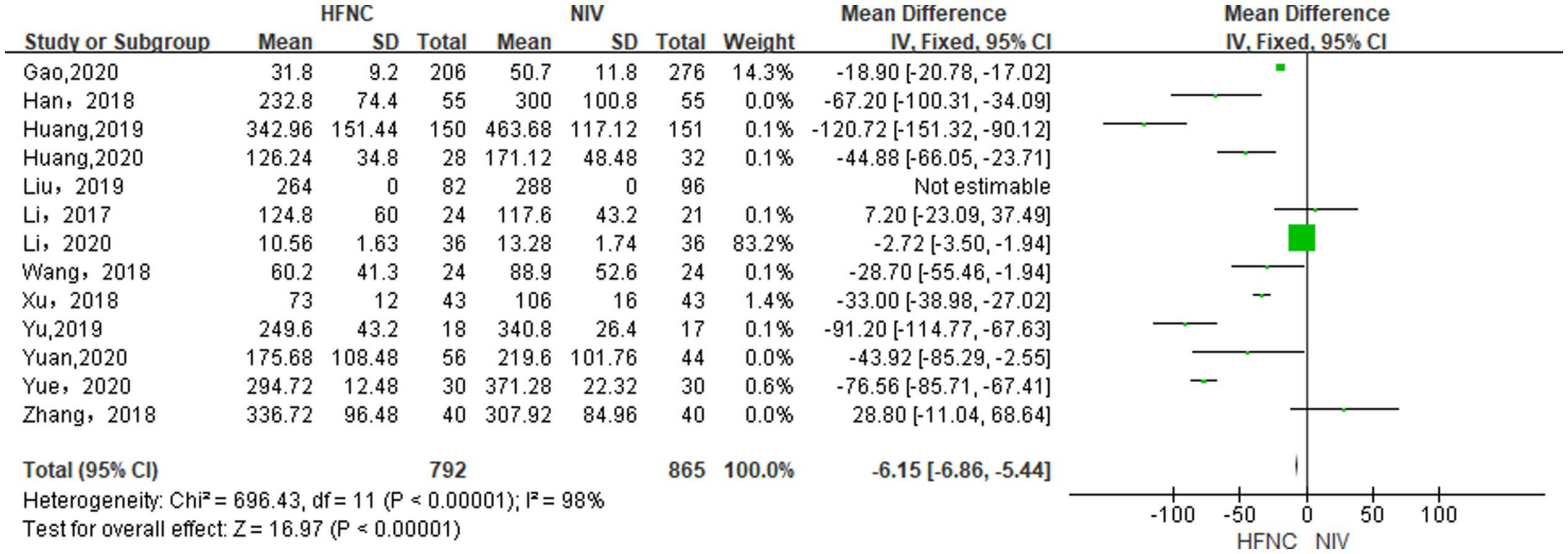
Forest plot of meta-analysis comparing the length of stay in ICU between HFNC group and NIV group.

With 13 RCTs (13–24, 27) included, analysis in a random-effects model (*p* < 0.00001, *I*^2^ = 98%) suggested that HFNC was superior to NIV in reducing length of stay in ICU (day) [MD = −6.15, 95% CI (−6.86, −5.44), *p* < 0.00001, Figure 7].

#### 3.3.7 Differences in the mortality rate between groups

**Figure 8:**
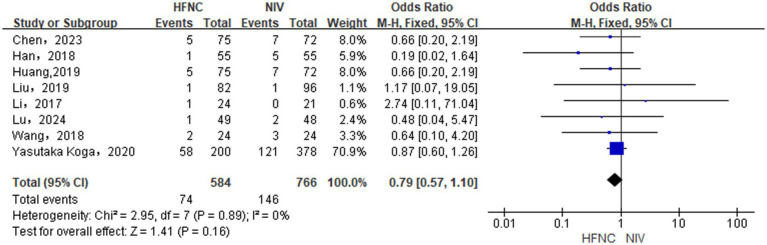
Forest plot of meta-analysis comparing the mortality rate between HFNC group and NIV group.

With the inclusion of 6 RCTs (17, 19, 23, 24, 27, 30) RCT and 2 NRCTs (31, 32), our analysis using a fixed-effects model (*p* = 0.88, *I*^2^ = 0%) observed that HFNC did not differ from NIV in reducing mortality [OR = 1.29, 95% CI (0.96, 1.72), *p* = 0.09, Figure 8].

#### 3.3.8 Results of sensitivity analysis

Meta-analysis of PaO_2_, PaCO_2_, OI levels, and length of stay in ICU showed high heterogeneity, while the source remained unclear as sequential exclusion of studies did not significantly reduce heterogeneity. Sensitivity analyses using a random-effects model confirmed the robustness of the results.

#### 3.3.9 Publication bias analysis

**Figure 9:**
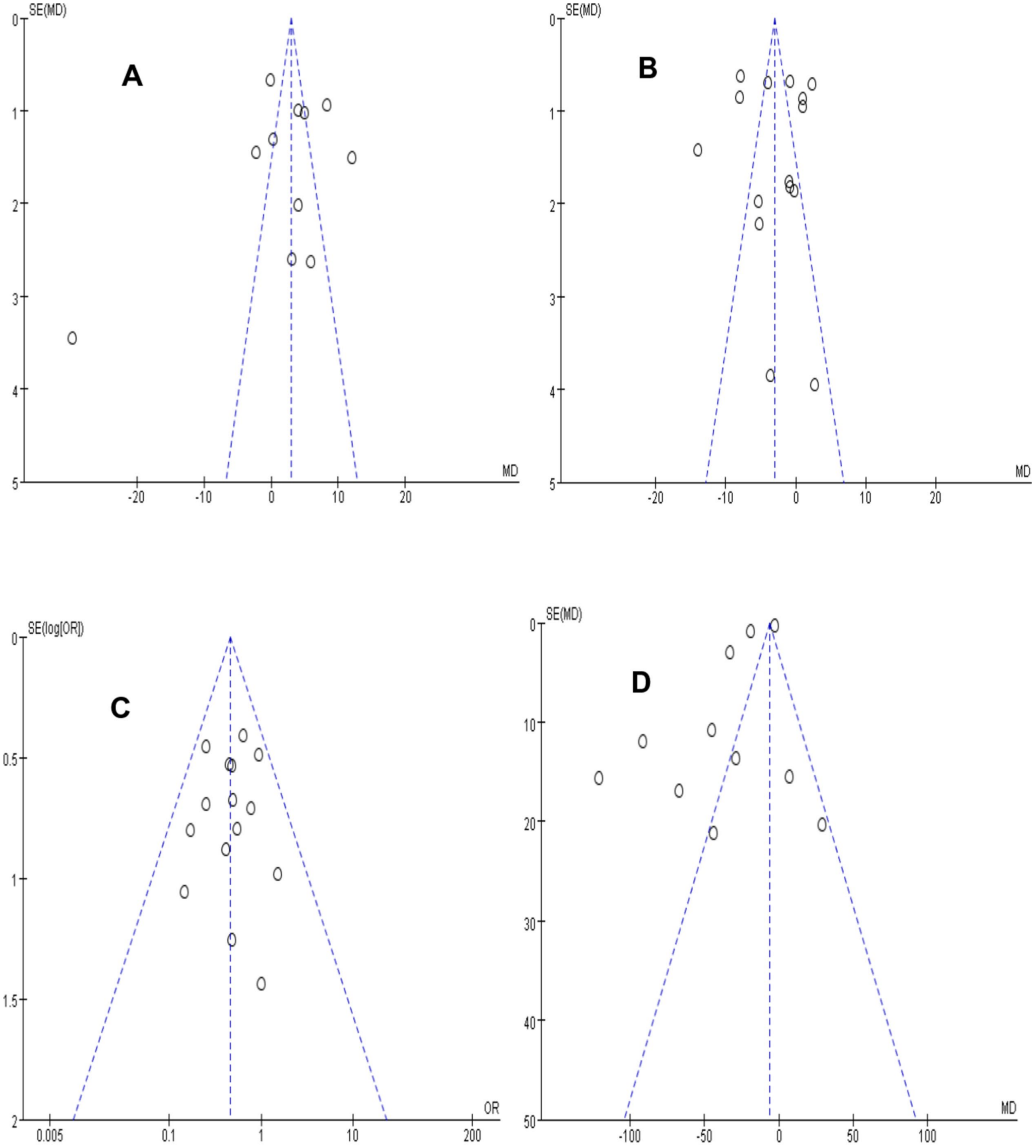
Funnel plots of outcome indicators **(A–D)**.

Funnel plots generated using RevMan indicated potential publication bias for PaO_2_ levels (Figure 9A) and length of stay in ICU time (Figure 9D), possible bias for PaCO_2_ levels (Figure 9B), and minimal bias for the re-intubation rate (Figure 9C). These findings highlighted the need for cautious interpretation and a more comprehensive literature search in future studies.

## 4 Discussion

Respiratory failure is a serious clinical syndrome caused by various diseases, which may induce significant respiratory dysfunction, and serve as a major cause of ICU morbidity and mortality (33). Patients often require invasive mechanical ventilation initially (34), with NIV occupying an important position in post-extubation recovery (35). However, NIV can cause facial pressure ulcers and discomfort (36), and it may impair ciliary function and airway defense owing to its limited warming and humidifying capabilities, restricting its clinical application (37).

HFNC, as a novel oxygen delivery device, demonstrates multiple clinical advantages. It can generate positive end-expiratory pressure (PEEP) by delivering temperature- and humidity-regulated high-flow gas. This can promote alveolar recruitment and gas exchange, effectively improving hypoxemia, particularly effective in patients at risk of alveolar collapse, such as those with respiratory failure. The PEEP effect may benefit the maintenance of alveolar patency, mitigating ventilator-induced lung injury from atelectasis and thereby enhancing oxygenation. Additionally, through flushing the upper airway dead space, HFNC can facilitate the improvement of CO₂ clearance efficiency, holding significant importance for patients prone to CO_2_ retention, as it can reduce PaCO_2_ and alleviate respiratory acidosis. Concurrently, HFNC preserves mucociliary function, decreases airway resistance, and reduces respiratory effort. By diminishing the burden on respiratory muscles, it may enhance the function of breathing for patients with compromised respiratory function, alleviating dyspnea and respiratory muscle fatigue as well (38).

The nasal cannula interface can avoid skin contact, thus eliminating facial pressure injuries and discomfort associated with traditional masks. It can also preserve patients’ ability to eat, speak, and perform daily activities—a critical advantage for those requiring long-term oxygen therapy, such as COPD patients during stable rehabilitation. Maintaining normal nutrition and communication can enhance patients’ quality of life and treatment adherence, while significantly improving comfort and reducing complications such as skin breakdown and infection caused by fixed interfaces (39–41). In addition, its integrated gas warming and humidification system can further improve patient tolerance and comfort, especially for those in dry/cold environments or with sensitive airway mucosa (42). HFNC has thus emerged as a viable alternative to NIV for managing severe hypoxemia (43).

With the inclusion of 20 articles (13–32) (*n* = 3, 159), this study evaluated the effectiveness of HFNC (*n* = 1,447) and NIV (*n* = 1,712) in post-extubation respiratory failure. The primary outcomes included PaO_2_, PaCO_2_, OI, re-intubation rate, pulmonary infection rate, length of stay in ICU time, and mortality, with a purpose to offer stronger evidence for clinical decision-making.

Consequently, HFNC outperformed NIV in improving PaO_2_ and OI, reducing PaCO_2_ and re-intubation rate, as well as shortening the length of stay in ICU, without difference in pulmonary infection or mortality. Similarly, Yue (21) noted that HFNC could deliver high-concentration, humidified oxygen, enhancing oxygenation and maintaining stable PaCO_2_, while avoiding complications such as abdominal distension and aspiration occurred in NIV; and it could also reduce the requirement for re-intubation and shorten the length of stay in ICU. Lin et al. (44) also highlighted the ability of HFNC to improve respiratory mucosal function, promote alveolar opening, and stabilize oxygen levels.

Wang et al. (24) documented that HFNC could improve oxygenation, comfort, and reduce invasive ventilation use compared to NIV, with better tolerance and lower risks of complications such as ventilator-induced lung injury and pneumonia. However, HFNC has limitations, including inconsistent airway pressure and challenges in patients with poor expectoration, such as post-lung transplant recipients, who may require bronchoscopy for sputum drainage. HFNC is safe and effective only if expectoration improves. Therefore, non-invasive ventilatory support should be tailored to individual needs, and combination therapies may enhance the therapeutic outcome for respiratory failure.

Our results indicate high heterogeneity between HFNC and NIV in PaO_2_, PaCO_2_, OI, and length of stay in ICU. Sensitivity and meta-regression analyses identified no influencing factors. Possible reasons may include differences in baseline characteristics; variability in treatment plans; treatment tolerance; study design and sample size limitations; patient compliance and nursing intervention frequency; as well as treatment timing and duration.

This study has certain limitations. Firstly, it investigated the short-term efficacy, with an absence of long-term follow-up. Future multi-center and large-sample studies should be conducted to verify the clinical efficacy of HFNC in sequential mechanical ventilation and explore long-term outcomes such as quality of life and survival rates. Secondly, funnel plot asymmetry for PaO_2_, PaCO_2_, length of stay in ICU, and re-intubation rates may suggest potential publication bias, which may compromise result reliability and clinical decisions. Future studies should emphasize pre-registration and transparency to minimize bias.

## 5 Conclusion

In summary, HFNC demonstrates superior efficacy over NIV across diverse patient populations and clinical conditions. HFNC can significantly improve key respiratory parameters—including PaO_2_, OI, and PaCO_2_ reduction—while also lowering re-intubation rates and shortening length of stay in ICU. Collectively, findings in the present study support the clinical adoption of HFNC as a valuable respiratory support alternative.
