# Correction: Meta-analysis of high-flow nasal cannula oxygen therapy versus non-invasive ventilation after invasive mechanical ventilation

**DOI:** 10.3389/fmed.2025.1712847

**Published:** 2025-10-15

**Authors:** Mailidan Maimaitiniyazi, Tuersunayi Yisimitila, Muyesaier Maimaitiniyazi, Meiheliya Maisuti, Ailifeire Aihaiti, Zhang Chenfei, Yilizhati Nijiati, Muyesai Nijiati

**Affiliations:** ^1^Emergency Center, People's Hospital of Xinjiang Uygur Autonomous Region, Ürümqi, China; ^2^Xinjiang Medical University, Ürümqi, China

**Keywords:** HFNC, NIV, invasive mechanical ventilation, meta-analysis, respiratory failure

Affiliation 2 “Xinjiang Medical University, Ürümqi, China” was omitted from the paper. This affiliation has now been added for authors Mailidan Maimaitiniyazi, Tuersunayi Yisimitila, Muyesaier Maimaitiniyazi, Meiheliya Maisuti, Ailifeire Aihaiti, and Zhang Chenfei.

The original version of this article has been updated.

